# The Hospital Officers' Association

**Published:** 1904-10-29

**Authors:** 


### THE HOSPITAL OFFICERS' ASSOCIATION.

This Association held its second annual dinner on Saturday, the 22nd inst., at the City of New York Restaurant. The President of the Association, Mr. Lewis H. Glenton-Kerr, was in the chair and was supported by Mr. J. Danvers Power as the guest of the evening and about 70 members and visitors.

Mr. J. Danvers Power replying to the toast of "The Hospitals," stated that a successful hospital was one of which it was said it was doing its best. Practically the officers of each institution were the only people who really knew all the circumstances and on them depended the future of London hospitals. What they required, however, were the advantages of co-operation and those they could not very well get for themselves without outside assistance. He thought that some one or other or all of the great collecting funds had it in their power to give help in this direction. Many questions, as for instance "Payments of Patients" and "Pensions for Officers" on which the practice now differed, might be competently and thoroughly investigated by specially appointed committees who would advise, the rest being left to the hospitals themselves. If the hospitals followed the advice given they would be presenting a united front to a disunited public. That would be co-operation and its advantages were apparent.

An excellent musical programme concluded a very enjoyable evening.

